# Microglia isolation from aging mice for cell culture: A beginner’s guide

**DOI:** 10.3389/fncel.2023.1082180

**Published:** 2023-01-19

**Authors:** Akshay Kumar Vijaya, Monika Iešmantaitė, Virginia Mela, Daiva Baltriukienė, Aurelijus Burokas

**Affiliations:** ^1^Department of Biological Models, Institute of Biochemistry, Life Sciences Center, Vilnius University, Vilnius, Lithuania; ^2^Department of Medicine and Dermatology, Faculty of Medicine, University of Malaga, Málaga, Spain

**Keywords:** microglia, isolation, Percoll, phagocytosis, senescence, chemotaxis, ROS

## Abstract

Microglia, the innate immune cell of the central nervous system, play significant roles in brain development, maintenance, homeostasis, and neuroinflammation. Although numerous methods have been developed to isolate microglia from embryonic or postnatal mouse brains, still major difficulties exist in isolating microglia from adult mice, often resulting in low yield and risk of cellular activation. Therefore, there is a need for a more efficient method to isolate pure and high-yield microglia from adult mice to study various neurodegenerative diseases. The aim of this study was to develop a fully functional protocol for the isolation of microglia by comparing different protocols. We investigated the efficacy of three protocols in terms of cell yield, purity, cellular activation, cellular aging, and migration properties and proposed the modified protocol (PROTOCOL 1), which provides an optimal yield of functional microglial cells with a minimum of material and equipment and allows young researchers with little experience to isolate microglia and helps them to delve deeper into the world of neuroscience.

## Introduction

Microglia are the resident macrophages in the central nervous system (CNS) (Bennett and Bennett, 2020). Their functions in a healthy brain are to manage synaptic connections, respond to infections and chemical or physical harm, and contribute to processes associated with removing dying neurons or cellular debris (Harry and Kraft, 2012). Microglia are also involved in synaptic pruning and remodeling in the brain. Any impairment of the CNS, including infection, trauma, or metabolic dysfunction, results in microglia activation. Following activation, microglia undergo morphological and functional changes (Saijo and Glass, 2011; Tang and Le, 2016; Simpson and Oliver, 2020). However, disrupted sentinel tasks and deregulation of the defense function in microglia can lead to neuronal damage (Hickman et al., 2018). Thus, aberrant microglial activation in the brain leads to neuroinflammation and neurodegeneration (von Bernhardi et al., 2015; Megur et al., 2020). Therefore, it is necessary to study these cells to understand their role in brain development and neuroinflammation. Primary cells are more relevant to biomedical research than the use of cell lines. Moreover, they have a number of applications, including understanding cellular morphology, functionality, cytokine production (Jolivel et al., 2021), scaffold research (Watson et al., 2017), and disease models (Stansley et al., 2012).

Nowadays, scientists often use microglial cell lines or primary microglia isolated from embryonic (Gingras et al., 2007) or neonatal animals (Floden and Combs, 2007) as *in vitro* microglial models. However, microglial cell lines are not always a preferred model because they express few, if any, genes characteristic of adult microglia (Butovsky et al., 2014), have a distinct transcriptome signature (Gerrits et al., 2020), and are phenotypically separate from primary microglia (Ni and Aschner, 2010). Furthermore, neonatal primary microglia are not fully mature and behave differently from adult microglia (Crain et al., 2013). Consequently, these *in vitro* microglia models might not be suitable for studying neurodegenerative diseases in which aging plays a crucial role. Research into the biology of the microglial cells is hampered by the difficulty in obtaining a sufficient quantity of primary microglial cells from old animals to routinely perform experimental techniques and elucidate signaling pathways (Gordon et al., 2011). For example, myelin, a multilayered, lipid-rich material that surrounds the axon, can interfere with downstream applications during cell culture and therefore need to be removed (Nikodemova and Watters, 2012). In addition, large numbers of animals are required to study cell function. Moreover, maintaining microglia in culture is associated with limited cell proliferation, which in turn can lead to a number of difficulties (Aktories et al., 2022).

In addition, cell yield is affected by the homogenization procedure and consideration must be given to whether enzymatic or mechanical digestion is used, as this could alter the expression of cell surface markers (Garcia et al., 2014). Several protocols have been developed for the isolation of microglia. Percoll density gradients are widely used to separate microglia (Cardona et al., 2006; Frank et al., 2006; Agalave et al., 2020). These gradients contribute to cell size separation (Frank et al., 2006) and effectively remove myelin and cellular debris (Grabert and McColl, 2018). The disadvantage of this technique is the long centrifugation and dissociation of brain tissue before spinning down, which can lead to excessive cell damage and low microglial yield (Ni and Aschner, 2010). In recent years, researchers have observed the emergence of new protocols, such as magnetic bead separation, which targets a specific marker using an antibody-magnetic bead conjugate (Podolnikova et al., 2016). Also, the protocol developed by Woolf et al. (2021), which takes advantage of the adherent property of microglia, has enabled a new method for isolating pure microglia. Removal of red blood cells is a critical step in the isolation of microglia when perfusion is not performed (Buenaventura et al., 2022).

As microglia are susceptible and reactive cells, different preparation protocols may result in slightly different phenotypes of microglial cells, which in turn can potentially influence experimental results.

The aim of the present study was to isolate microglia using three different protocols, characterize the cells based on cell purity, functionality, and cellular aging, and validate our redesigned protocol (PROTOCOL 1) (Bordt et al., 2020). We have described an efficient method for isolation of microglia from adult mouse brain that allows assessment of microglial activities *in vitro* that reflects their functions *in vivo*. We modified the existing protocol to provide a simpler and faster method for isolating large numbers of microglia with relative high purity using common laboratory reagents. To prove our concept, we compared isolation procedures and post-isolation characterization.

## Materials and methods

### Animals

For this study, 34 adult (6-month-old) C57BL/6J mice obtained from Janvier Laboratories, France, were used the permission of the Lithuanian State Food and Veterinary Service to perform the experiment was given (No. G2–104), and the maintenance and experiments complied with the requirements of 2010/63/EU Directive. Animals were housed under controlled conditions (22°C ± 1, humidity 40%, food and water were supplied *ad libidum*) and under veterinary supervision. The animals were euthanized using cervical dislocation.

### Isolation and maintenance of microglial cells

Brains were removed from euthanized animals as quickly as possible and kept cold (+4°C) in a medium containing antibiotics for the further procedure (as described in Supplementary material). The average brain weight collected from this age group of mice was approximately 500 mg. Dissection of tissue must be rapid to prevent cell death and activation. Tools and reagents must be sterile and prepared in advance. Careful in preparing the right pipette size.

**FIGURE 1:**
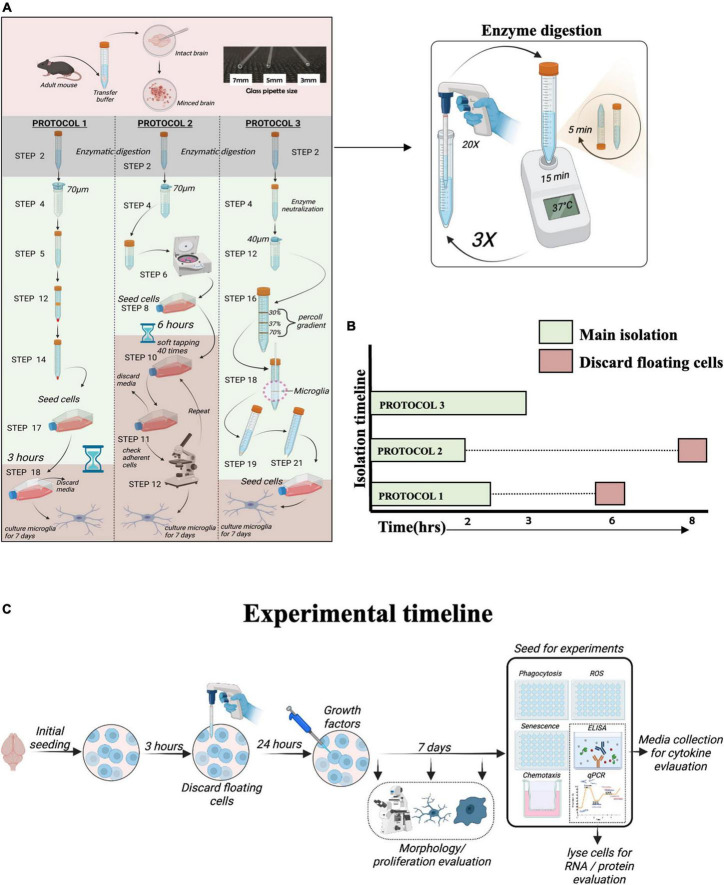
Overview of protocols for microglia isolation. **(A)** Schematic representation of the protocols used to isolate adult mouse microglia. **(B)** The total duration of isolation for each protocol. **(C)** Schematic diagram of experimental timeline.

Isolation of microglia was performed using three different protocols: PROTOCOL 1 (modified by our laboratory), PROTOCOL 2 (Woolf et al., 2021), and PROTOCOL 3 (Lee and Tansey, 2013), as described in the Supplementary material. Figure 1 depicts a schematic diagram of step-by-step isolation procedures. Cells were seeded in a T25 culture flask in 50% Dulbecco’s Modified Eagle Medium/Nutrient Mixture F-12 (DMEM/F-12; ThermoFisher Scientific, Lithuania) with GlutaMAX™ supplement (ThermoFisher Scientific, Lithuania) and 50% conditioned medium collected from mixed cells isolated from the brain (as described in Supplementary material) with 10% Fetal bovine serum (FBS) and 1% penicillin/streptomycin (10,000 units). On day 2, the medium was supplemented with macrophage colony-stimulating factor (M-CSF; 100 ng/mL; R&D Systems, UK) and granulocyte-macrophage colony-stimulating factor (GM-CSF; 100 ng/mL; R&D Systems, UK). Microglia were cultured for 7 days, and the medium was changed every 3 days. 7 days is necessary for the isolated microglia to recover their sub-reactive morphology. During the 7-day adaptation period, cells were monitored every day and imaged on day 1, day 4, and day 7 to show the developing morphology. Images were taken under a light microscope at 20x magnification, as shown in Figure 3. After 7 days in culture, cells were counted with a trypan blue exclusion assay using a hemocytometer. Additionally, upon attaining morphology and confluency, cells were detached from flasks using 0.05% trypsin and 0.5 mM EDTA and seeded into 48 well plates for experiments. The flasks were not coated with any material for culturing as well as experimentation.

**FIGURE 2:**
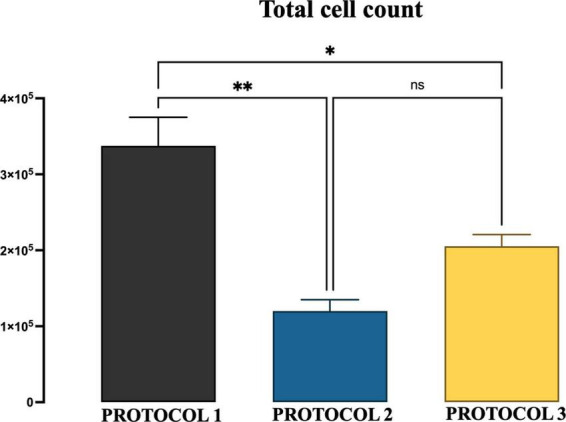
The yield of microglial cells obtained with three different protocols. The graphs show the group means of the number of microglial cells per mouse brain obtained from 6-month-old mice by the isolation procedure of PROTOCOL 1, PROTOCOL 2, and PROTOCOL 3. For statistical analysis, the one-way ANOVA was used [*F*_(2,4)_ = 20.60; *p* < 0.0078], and a subsequent *post-hoc* test was carried out using Tukey’s comparison test: *p < 0.05; ^**^p < 0.01; *N* = 3.

**FIGURE 3:**
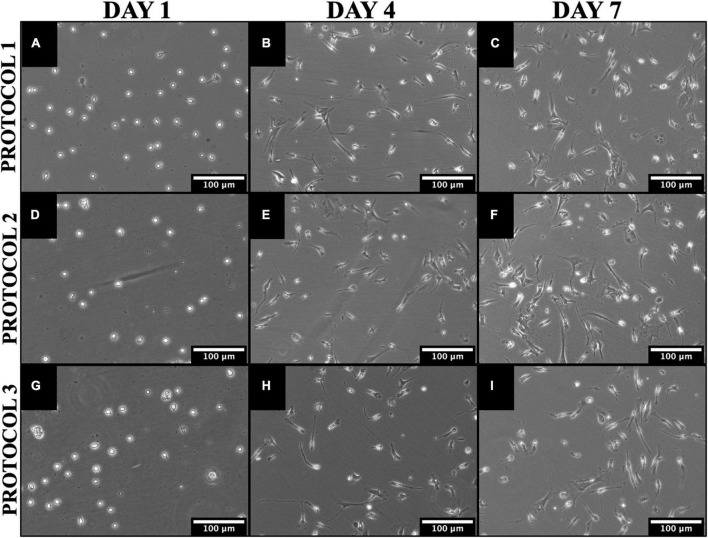
Morphological and proliferative differences between cells isolated by different methods. Representative phase-contrast images of mouse microglia cultures over 7 days in culture illustrating significant growth time points for PROTOCOL 1 **(A–C)**, PROTOCOL 2 **(D–F)**, and PROTOCOL 3 **(G–I)**. Scale bars: 100 μm.

### Microglia purity assessment

Microglia were identified by their specific surface expression profile of CD11b using immunocytochemistry. After 7 days of culture, 100,000 cells/well were seeded in 48-well plates for immunocytochemistry analysis. Twenty-four hours after seeding, cells were fixed with freshly prepared 4% paraformaldehyde in PBS for 15 min. Cells were stained with CB11b antibody (1:150; PE, ThermoFisher Scientific; Lithuania), and nuclei were counterstained with DAPI dye. Fluorescence was visualized using a fluorescence microscope. Analysis was performed using ImageJ software. Multiple CD11b+ cells were counted, and the percentage was plotted.

### Assessment of microglia morphology

We evaluated cell soma size, the number of processes, and the roundness of the cells. It is known that activated microglia have bigger soma size, tend to be on the rounder side, and has fewer processes (Hovens et al., 2014). Morphological characteristics of the microglia were analyzed by using the image analysis software ImageJ. The area size and cell roundness were measured by using freehand selection. Area value determines how many pixels there are in the cell area. The more pixels are in the cells, the bigger it is. The rounder the cell, the more value in “Round” became closer to 1. Cell processes were counted by eye.

### Evaluation of TNF-alpha gene expression

To assess gene expression of TNF alpha in microglia cells after treatment with LPS, we performed qRT-PCR using QuantStudio™ 5 Real-Time PCR System (Thermo Fisher Scientific, USA). Total RNA was extracted using a High Pure RNA Isolation kit (Macherey-Nagel, Germany) according to the manufacturer’s protocol, and subjected to cDNA synthesis using a High-Capacity cDNA Reverse Transcription Kit (Thermo Fisher Scientific, USA). qRT-PCR for TNF-α was performed using their respective primers. Primer sequences were as follows: TNF-alpha, F: 5′-ATGGCCTCCCTCTCATCAGT-3′, R: 5′- TTTGCTACGACGTGGGCTAC-3′. Alpha tubulin was used as a housekeeping control gene, F: 5′-TGTGGATTCTGTGGAAGGC-3′, R: 5′-ATGAAAGCACACATTGCCAC-3′. Standardization was performed using Alpha tubulin and then fold increase was calculated keeping control group as 1 (arbitrary unit) in all protocols.

### Quantitation of cytokine TNF-alpha in cell culture supernatants

ELISA kits for the measurement of mouse cytokine TNF-alpha levels in cell culture supernatants were used (Thermo Fisher Scientific, USA). ELISA kits are based on the sandwich immunoassay technique. Supernatants were used diluted 2X. All procedures were performed according to the manufacturer’s protocols. In the last step, 3,3′,5,5′-tetramethylbenzidine (TMB) substrate solution was added to each well. The plates were monitored for 15 min for color development, the reaction in wells was stopped with 3.6% H2SO4 solution, and the wells were read at 450 nm with reference wavelength at 570 nm plate reader Varioskan Flash (ThermoFisher Scientific, Finland). A standard curve was generated from cytokine standard, and the cytokine concentration in the samples was calculated.

### Evaluation of the phagocytic properties of microglia

The phagocytic capacity of the microglia was determined by the uptake of 1 μm fluorescent latex beads (Sigma Chemical Co., USA). 100,000 cells/well were seeded in 48-well plate. Cells were divided into two groups: cells treated with LPS (10 ng/mL), and untreated cells. After 24 h, both treated with LPS, and untreated cells were incubated with 0.025% (w/w) fluorescent latex beads for 4 h at 37°C and 5% CO_2_. Cells then were rinsed twice with PBS, fixed with freshly prepared 4% (w/v) paraformaldehyde in PBS, and permeabilized (PBS + 1% Triton X-100). Cells were blocked for 30 min in PBS containing bovine serum albumin (3% BSA) and fetal bovine serum (10% FBS) and incubated for 1 h at room temperature with a primary conjugated antibody (anti-CD11b; 1:150; PE, ThermoFisher Scientific; Lithuania) in blocking solution. Nuclei were counterstained with DAPI dye (5 μg/mL), and fluorescence was visualized using a fluorescence microscope. Analysis was performed using ImageJ software and GraphPad software. The percentage of CD11b + cells with ingested latex beads > 10 was analyzed for treated and untreated cells.

### Assessment of microglial cell senescence

Microglial senescence was evaluated by determining the activity of β-galactosidase. 100,000 cells/well were seeded in a 48-well plate. After 24 h, senescence-associated β-galactosidase (SA-β-gal) activity was assessed using the Senescence Cells Histochemical Staining Kit (Sigma-Aldrich, USA) according to the manufacturer’s instructions. Briefly, cells were fixed (7 min in fixative solution), washed, incubated with β-galactosidase staining solution (overnight, 37°C), and visualized under a light microscope (Olympus IX51, Japan) using 20X magnification. Analysis was undertaken with ImageJ software. The number of β-galactosidase positive microglia was assessed and expressed as a % of the total number of cells.

### Evaluation of the migration properties of microglia

Chemotaxis was assessed by plating the cells (100,000 cells) on the Transwell insert in a Boyden chamber (pore size 8 μm; Corning Inc., UK). After 24 h, the medium in the chamber was replaced with a serum-free medium, while the bottom of the well-contained a medium with serum. Cells were incubated for 6 h and then stained with crystal violet. Elution buffer (0.1% acetic acid in 50% ethanol) was used to solubilize the crystal violet dye. The absorbance at 570 nm was measured by plate reader Varioskan Flash (ThermoFisher Scientific, Finland). The amount of crystal violet dye is directly proportional to the migratory cells trapped on the bottom side of the chamber. Analysis was performed by representing control as 1 (arbitrary unit) in all protocols and comparing the fold increase.

### Assessment of microglial oxidative stress (ROS)

To assess ROS, 100,000 cells were seeded into a 48-well plate. ROS generation was assessed by treating cells with LPS (10 ng/mL) for 24 h and staining with CellRox Deep Red (Thermo Fisher, USA) according to the manufacturer’s instructions. Briefly, CellRox Deep Red solution was added to the media following treatment and incubated in the dark for 30 min at 37°C, 5% CO_2_. Cells were then washed and fixed with 4% paraformaldehyde for 15 min. The nuclei were counterstained with DAPI dye (5 μg/mL), and fluorescence was visualized using a fluorescence microscope (Olympus IX51, Japan) at 20x magnification. Analysis was performed using ImageJ software.

### Statistical analysis

Data are reported using ANOVA with mean ± SEM, and the number of experiments is indicated in each case. Only chemotaxis was presented as a violin plot. A subsequent *post-hoc* test was carried out using Tukey’s comparison test. The significance level was set at *p* < 0.05. Graphpad Prism was used for statistical analysis (Graphpad Prism version 9.3.1, USA).

## Results

### Cell yield and morphological analysis

A high number of primary cells is critical for the study of microglia biology. Therefore, the starting point of this study was the evaluation of microglial cell yield obtained using three different protocols. We chose to compare isolation efficiency with our optimized protocol based on enzymatic dissociation and debris removal (referred to as PROTOCOL 1), with a technique that exploits the adherent properties of microglia (referred to as PROTOCOL 2), and with a method that uses a Percoll gradient (referred to as PROTOCOL 3). The total number of cells was determined by trypan blue exclusion after culturing the microglial cells for 7 days and expressed as a number of microglial cells per mouse brain. PROTOCOL 1 consistently produced a higher yield with an average of 340,000 cells compared to 100,000 (*p* < 0.0069) and 215,000 (*p* < 0.0288) in PROTOCOL 2 and PROTOCOL 3, respectively (Figure 2).

Under physiological conditions, microglia are characterized by a small cell body and predominating ramified morphology. However, cell activation triggers a morphological transformation from a ramified to an amoeboid-like, we evaluated the morphology of microglia after different isolation techniques to determine whether the isolation method can cause drastic cell activation *in vitro* and affect the proliferation rate. All microglia cultures showed similar morphological patterns during the culture phase for all isolations performed (Figure 3). After isolation, the cells had a round morphology. After 2–3 days, microglia started to gain their characteristic morphology (Figures 3B, E, H). After 7 days, the microglia have adapted to a ramified morphology (Figures 3C, F, I). In addition, qualitative assessment of the cell proliferation rate showed that cells from all isolations grew uniformly well over the 7 days. Thus, the morphology and growth rate was independent of the isolation method.

### Characterization of culture purity

**FIGURE 4:**
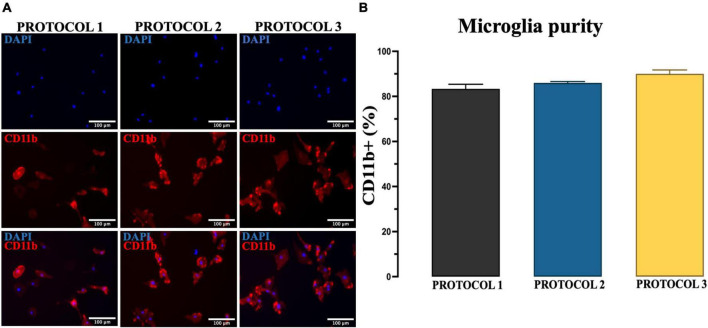
Evaluation of the purity of microglia. **(A)** Purity of cultures established by PROTOCOL 1, PROTOCOL 2, and PROTOCOL 3 was determined *via* immunocytochemistry using a specific marker for microglia (CD11b, red) and nuclear dye (DAPI, blue). Scale bar: 100 μm. **(B)** Graphical representation of the percentage of CD11b + cells at day 7 after isolation. Data are presented as mean ± SEM; *N* = 3.

Another essential requirement for the isolation method is the purity of the culture because foreign cells can overgrow microglia due to their higher proliferative capacity. Therefore, we evaluated whether different isolation methods can affect the purity of microglia culture by performing immunofluorescence staining with CD11b antibody (Figure 4A) and confirmed that the isolated primary microglia were 80–90% CD11b^+^. One-way ANOVA revealed no significant difference among protocols in the percentage of CD11b^+^ cells [*F*_(2,6)_ = 4.537; *p* = 0.0631] (Figure 4B). Thus, we demonstrated that all protocols might generate high purity microglia cell cultures from adult mice brain.

### Assessment of microglia morphology

**FIGURE 5:**
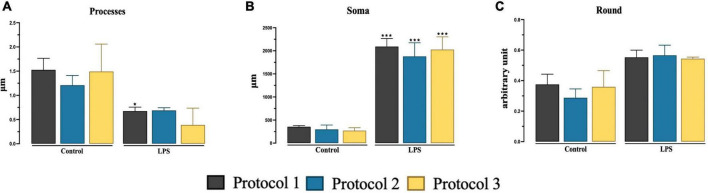
Evaluation of the morphology of 7-day cultured microglia and 7-day cultured stimulated microglia. Graphical representation of number of processes **(A)**, size of cell soma **(B)**, and roundness **(C)** at day 7. Data are presented as mean ± SEM; *N* = 3. ***p < 0.001 and *p < 0.05 compared to the control groups.

We determined the microglia state by morphological analysis of soma size (Ceyzériat et al., 2018), number of processes, and roundness of the cell (Fernández-Arjona et al., 2017). We were able to analyze the data sufficiently to allow discrimination between 7-day cultured microglia and 7-day cultured stimulated microglia. The number of processes was significantly higher in the PROTOCOL 1 control group compared to the LPS treated reactive cells and two-way ANOVA was used [*F*_(1,12)_ = 33.52; *p* < 0.001] (Figure 5A). Two-way ANOVA used [*F*_(1,12)_ = 372.0; *p* < 0.001] was used to analyze size of cell soma where reactive group showed significantly larger cell soma than control group (Figure 5B). Two-way ANOVA [*F*_(1,12)_ = 47.70; *p* < 0.001] showed no significant difference in all isolations in both groups (Figure 5C). Subsequent Tukey’s *post-hoc* test was done for all analysis.

### TNF-alpha gene expression

**FIGURE 6:**
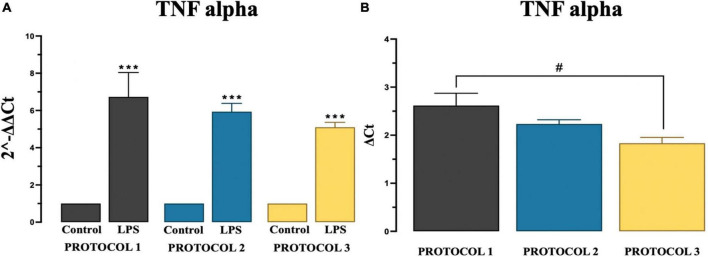
Evaluation of TNF alpha gene expression. **(A)** Graphical representation of TNF-alpha showing a significant fold increase in expression in LPS groups compared to control groups. **(B)** Comparison of TNF alpha gene expression among protocols after LPS treatment. Data are presented as mean ± SEM; *N* = 3. ***p < 0.001 compared to the control groups. PROTOCOL 1 shows a higher gene expression than PROTOCOL 3, #*p* < 0.05 for LPS treated groups.

There are overwhelming evidences proving chronic microglia reaction and overproduction of pro-inflammatory cytokine tumor necrosis factor alpha (TNF-α) (Brás et al., 2020). At basal levels, TNF-α has an important role in brain development and immune cell mediated inflammation (Perry et al., 2002). However, in certain pathological conditions, increased levels of this cytokine over activate microglia, which then causes neuronal damage, such as demyelination and/or neuronal degeneration. Overactivated microglia release cytotoxic molecules, including TNF-α, which propagate pro-inflammatory signatures (Muhammad, 2019). In our experiment, significant difference was observed in pro-inflammatory TNF-α gene expression in LPS stimulated cells in all protocols compared to control group as described in Figure 6A using two-way ANOVA [*F*_(1,12)_ = 330.9; *p* < 0.001] and subsequent Tukey’s *post-hoc* test. Amongst protocols, we observed a significant difference only between PROTOCOL 1 and PROTOCOL 3 as described in Figure 6B, One-way ANOVA was used to analyze data [*F*_(2,6)_ = 5.262; *p* < 0.05] and subsequent Tukey’ *post-hoc* test was done.

### TNF-alpha secretion

**FIGURE 7:**
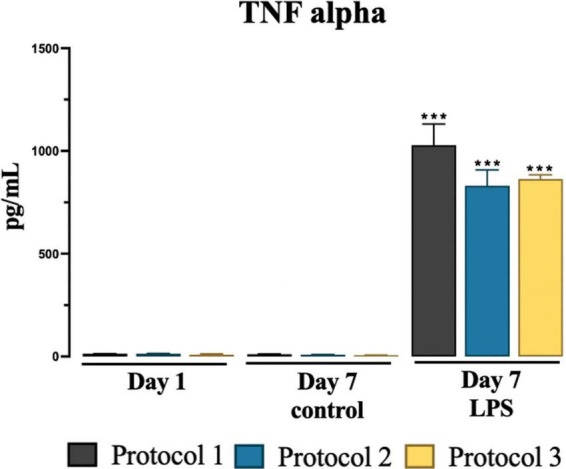
Evaluation of cytokine TNF-alpha secretion. Graphical representation of TNF-alpha conc. at day 1, day 7 for control and LPS treated cells. Data are presented as mean ± SEM; *N* = 3. ***p < 0.001 compared to the control day 7.

We determined no significant difference was observed in pro-inflammatory TNF-α levels in the collected media among isolation protocols at day 1 and day 7 controls and LPS treated groups. However, significant increase was observed in LPS treated groups at day 7 in all protocols compared to day 7 control groups as shown in Figure 7. For statistical analysis, two-way ANOVA was used [*F*_(2,12)_ = 245.9; *p* < 0.001] and subsequent Tukey’s *post-hoc* test.

### Comparison of the activation potential of microglia

It is crucial to understand the impact of the isolation procedure on microglia cells because the isolation itself could activate microglia, leading to false-positive results. Therefore, we studied the reactive microglia after isolation in terms of phagocytosis, oxidative stress, and migration.

#### Phagocytosis

**FIGURE 8:**
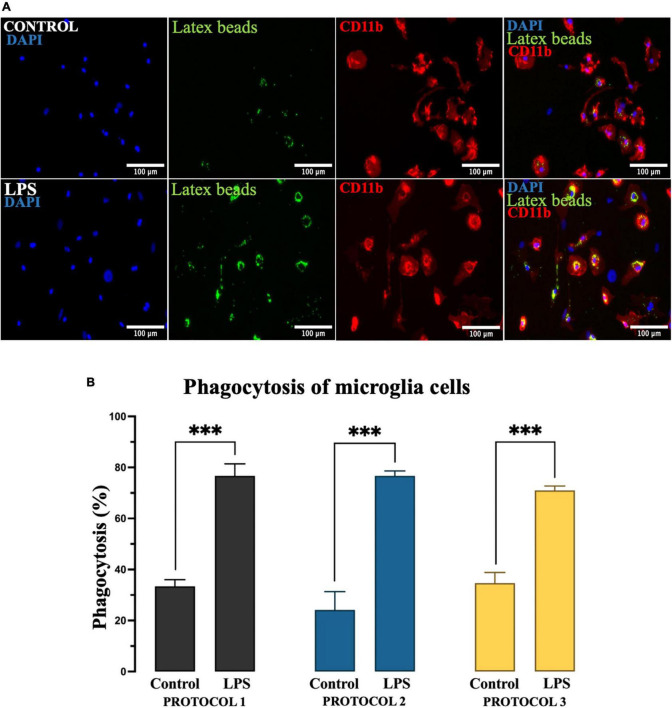
Evaluation of the phagocytic activity of microglia. **(A)** Representative image of microglia incubated with fluorescent latex beads (green), immunolabeled for CD11b to identify the cell soma (red), and nuclei stained with DAPI (blue). Scale bar: 100 μm. **(B)** The percentage of microglia involved in phagocytosis as the mean of cells that ingested at least 10 beads. Data are presented as mean ± SEM of the percentage of CD11b + latex beads + cells. ^***^p < 0.001.

Microglia exhibit phagocytic properties in response to inflammatory stimuli such as LPS. To evaluate the phagocytic activity of the isolated cells based on the protocols, we divided the established primary microglia into two groups – untreated and treated with LPS. After 24 h, cells were incubated with latex beads for 4 h, followed by immunocytochemical analysis (Figure 8A). For statistical analysis, two-way ANOVA was used [*F*_(1,12)_ = 501.7; *p* < 0.001] and the subsequent Tukey’s *post-hoc* test showed that phagocytic activity was dramatically increased in the presence of LPS. However, the phagocytic properties of microglia were not affected by different isolation protocols in both treated and untreated cell populations (Figure 8B).

#### Migratory capacity of microglia

**FIGURE 9:**
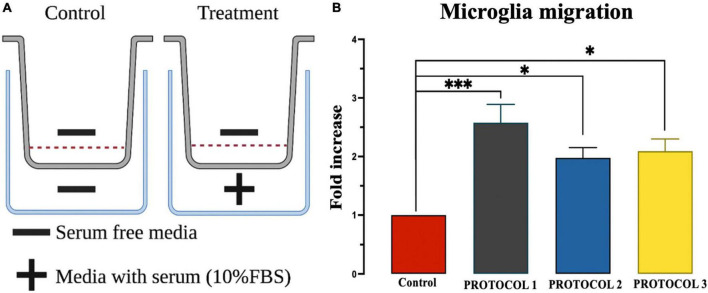
Evaluation of microglia migration. **(A)** Overview of chemotaxis setup. **(B)** Migration was assessed using chemotaxis chambers and analyzed by UV spectroscopy at 570 nm wavelength. Results were expressed as calorimetric intensity of the migrating cell compared to control cells. Data are presented as mean ± SEM; ***p < 0.001 and *p < 0.05 compared to the control.

Microglial cells are highly mobile under inflammatory conditions, which is one of the crucial properties of the immune response. Therefore, it is important to check the mobility of microglia after isolation. To examine the migratory function of microglia, chemotaxis was performed in control cells (without serum) and tested cells (with serum), as shown in Figure 9A. One-way ANOVA showed no detectable change in migration properties in either of the isolation protocols (Figure 9B). However, FBS resulted in higher cell movement compared to the control.

#### Microglia and ROS

**FIGURE 10:**
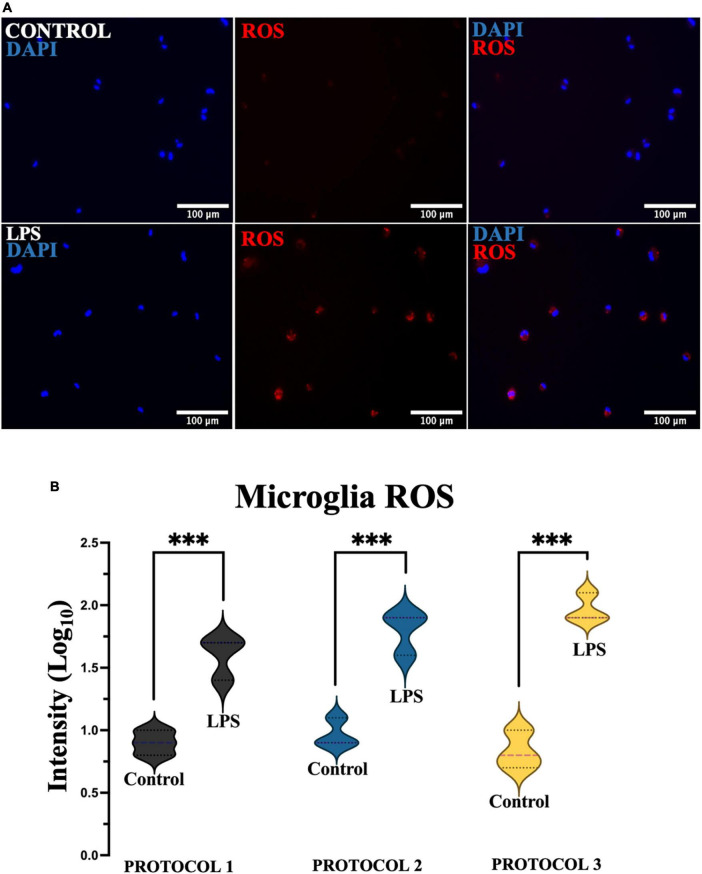
Evaluation of oxidative stress. **(A)** Representative images of microglia (PROTOCOL 1) exhibiting ROS (red) and nuclei stained with DAPI (blue). Scale bar: 100 μm. **(B)** Fluorescence intensity was measured, normalized, and normalized to log_10_. Data are presented as a violin plot. ^***^p < 0.001.

Reactive oxygen species play a key role as cellular defense mechanisms, therefore it is crucial to determine ROS production after isolation. To assess oxidative stress, cells were divided into 2 groups, untreated and treated (LPS) to induce ROS production (Figure 10A). Two-way ANOVA was used for statistical analysis [*F*_(1,12)_ = 177.8; *p* < 0.001], and the subsequent Tukey’s *post-hoc* test showed a steep increase in the production of ROS in the LPS-treated cells, however, no difference was detected between the isolation protocols in the treated and untreated cells (Figure 10B).

Consequently, it can be concluded that the isolation procedures did not affect microglia reaction. In all cases, primary microglia were more sensitive to LPS treatment.

### Aging of the microglia

**FIGURE 11:**
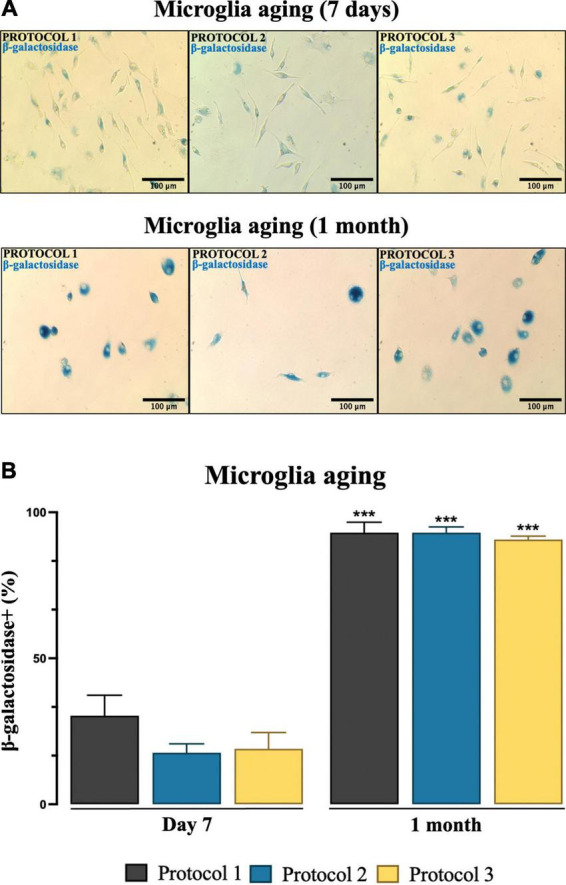
Estimation of microglia senescence over an extended period of time. **(A)** Representative images of microglia showing SA-β-gal activity (blue) from different isolation protocols. Images were taken under a light microscope at 20x magnification. Scale bar: 100 μm. **(B)** SA-β-gal-positive cells were counted and results were expressed as mean ± SEM; ***p < 0.001, compared to the day 7 groups.

It is important to evaluate cellular senescence as it is related to cell dysfunction. To assess cellular senescence at day 7 (when well-defined morphology is achieved) and progression over 1 month (Figure 11A) after isolation, SA-β-gal activity was analyzed. ANOVA for microglia after 7 days [*F*_(2,6)_ = 4.862; *p* = 0.0556] revealed no statistically significant difference, yet microglial cells isolated with PROTOCOL 1 showed a tendency to express more β-galactosidase (*p* = 0.0556) (Figure 11B). Senescence was rapidly increased *in vitro* for all isolation protocols after 1 month [*F*_(2,6)_ = 0.8909; *p* = 0.4584], however, no difference in senescence was observed between the different isolation protocols (Figure 11B). Differences in cellular morphology were observed between day 7 (specific ramified morphology) and 1 month [round senescent specific morphology (Figure 11B)].

## Discussion

Microglia serve as critical sensors, effectors, and regulators of CNS homeostasis during development, health, and disease (Helmut et al., 2011; Rubio-Araiz et al., 2018; Megur et al., 2020). Even in a healthy brain, microglia are highly active, pulling in and out motile processes through which they monitor their microenvironment and dynamically interact with surrounding cells (Parkhurst and Gan, 2010; Hanamsagar and Bilbo, 2017). Increasing knowledge now suggests that microglia have multifactorial effects that extend far beyond their traditional role in immunity. Thus, the study of microglial mechanisms is critical to broad areas of neuroscience.

With available primary microglia monocultures, many different assays are possible, such as microglia activation studies and screening for biomarkers and metabolites (Telpoukhovskaia et al., 2020). Primary microglia cultures mimic the host system and perform better in functional assays compared to microglia cell lines (Luan et al., 2022). Unique research in the field of genetically modified animals and transgenic disease models for neurodegenerative diseases is possible and can be extended to primary cell cultures to study and understand these unique systems. In addition, these systems can be studied in variety of species models to compare microglial functions (Montilla et al., 2020) and characterize microglia across species. Primary cell cultures are essential for certain research and have a number of applications. Researchers use cell cultures for various cell types, e.g., cardiac cells (Avazzadeh et al., 2022), nerve cells (Wei et al., 2022), hepatocytes (Miyoshi et al., 2022) etc., and applications such as understanding cell morphology (Giulian and Baker, 1986), functionality (Stansley et al., 2012), stimulated cytokine production (Bokarewa et al., 2021), scaffold research (Lizana-Vasquez et al., 2022), and model systems (Law et al., 2021). These aspects can be studied and visualized more reliably by cell culture techniques than by protein and RNA expression analyzes. By obtaining a high yield of cells, researchers could perform a range experiments from cell culture to RNA/protein work, which would allow them to take a multidiscipline approach to their hypothesis.

Nevertheless, microglia cell culture requires care and attention to details. Primary microglia cell culture systems are highly susceptable to various factors including the choice of coating for the culture flasks or the supplements in the media itself such as serum, growth factors, or added metabolites. Specific media must be used, DMEM f12 with GlutaMAX and supplemented with various necessary growth factors such as M-CSF and GM-CSF, which are important for microglial differentiation (Ginhoux et al., 2010; Wang et al., 2012) to promote growth and survival of the culture (Bohlen et al., 2017). In addition, experiments must be conducted within specific time frame, as the culture lifetime of microglia is short due to rapidly progressing senescence. Microglia-like cells that naturally develop in organoid cultures exhibit a typical ramified morphology that is much more similar to the *in vivo* morphology compared to other cell culture systems (Aktories et al., 2022). One of the unique properties of microglia is that they change shape depending on the microenvironment (Saijo and Glass, 2011; Candlish and Hefendehl, 2021). It becomes even more complicated when we further divide them into their sub-states (Tang and Le, 2016; Candlish and Hefendehl, 2021). Purity of cell culture is essential for cell growth and proper assessment of microglial functions. Other cell types, particularly fibroblasts, can become dominant and take over the population, affecting (i) microglial proliferation and (ii) their response to stimuli. Comparison of the protocols showed no difference in the purity of the cells. We were able to achieve a purity of microglia culture of more than 80% with all methods.

Our work primarily intended to help researchers who would like to work with cells *in vitro*.

We modified a protocol developed by Bordt et al. (2020) based on enzymatic dissociation and debris removal (PROTOCOL 1). Our modification allowed us to avoid the use of magnetic beads. Since microglia have strong adherent properties, they are the first cells to attach to the plastic. Instead of separation by magnetic beads, we removed all unwanted and non-adherent cells 3 h after isolation. The efficacy of this isolation procedure was compared with a technique that uses the adherent properties of microglia (PROTOCOL 2) (Woolf et al., 2021) and with a method using the Percoll gradient (PROTOCOL 3) (Lee and Tansey, 2013).

One of the critical parameters is the duration of the protocol to isolate the cells. The longer the isolation takes, the higher the likelihood of cell death (Chen et al., 2017), handling errors, and possible alteration of gene expression. Therefore, it is important to mention that PROTOCOL 3 is time-consuming (it takes about 4 h to perform) and requires a high level of expertise. On the other hand, PROTOCOL 2 requires only 1 h of hands-on work and is comparatively straightforward but has a long incubation period prior to myelin removal that may affect cell viability (Nikodemova and Watters, 2012). In comparison, PROTOCOL 1 requires 3 h of hands-on work but yields the highest number of cells. In addition, this technique removes myelin, which may affect the ability to isolate cells.

Microglia nomenclature is important in order to prevent misinformation to young researchers (Paolicelli et al., 2022). As a result, we will be referring to 7-day cultured microglia as sub-reactive microglia (homeostatic-like cells) and 7-day stimulated microglia as reactive microglia. In our work, we highlighted the change in microglial morphology after isolation in a 7-day (Figure 3), during which the cells acquired their specific phenotype. We observed the typical ramified morphology consistent with other studies (Glenn et al., 1992; Ling and Wong, 1993). When comparing the protocols, no change in phenotype was observed in all of them. Thus, all protocols result in a similar ramified morphology suitable for experimental work. We determined cellular morphology based on cell soma, cell processes, and rounding. LPS induced cellular reaction is observable through morphology changes. Reactive microglia are rounder, have a larger soma and fewer processes compared to sub-reactive microglia (Leyh et al., 2021). We examined the morphology after 7 days of isolation to determine if the cells are suitable for experiments.

Using all protocols, we were able to culture sub-reactive microglia, compared to the reactive morphology exhibited by the LPS-stimulated group, as shown by morphology measurements. TNF-alpha is upregulated in pro-inflammatory immune responses and its signature is widely used as a biomarker. LPS-stimulated microglia exhibit a pro-inflammatory state in which they secrete the cytokine TNF-alpha (Lively and Schlichter, 2018). We studied TNF-alpha cytokine secretion and gene expression for pro-inflammatory signatures 7 days after isolation and found that the isolation protocol had no effect on cellular activation as evidenced by the upregulation of both gene and cytokine expressions in the LPS-treated groups. Therefore, we are confident that our protocol is capable of isolating unreactive microglia and is suitable for functional experiments.

Microglia are considered the professional phagocytes of the CNS, a function that is critical in brain development and pathology as well as regeneration (Helmut et al., 2011). The isolation itself could activate microglia, which could lead to false-positive results. Therefore, the isolation protocol used to obtain these cells should minimize artificial activation. Various pathological insults are used to activate microglia *in vitro*. Recent evidence suggests that microglia, when exposed to inflammatory stimuli such as LPS, switch their metabolism to glycolysis (Holland et al., 2018; Rubio-Araiz et al., 2018) and exhibit an inflammatory phenotype (Holland et al., 2018; Mcintosh et al., 2019), followed by marked phagocytosis. In our study, the isolation protocols showed no difference in phagocytic activity with or without the introduction of LPS. However, isolated cells with all three protocols without LPS showed significantly lower phagocytosis potential than LPS-treated cells. Therefore, all protocols can generate unreactive microglia.

In response to pathological stimuli, microglia act as sensors of brain injury, become reactive, and migrate to the site of injury to clear damaged cells and cellular debris by phagocytosis (Gyoneva et al., 2009). To mimic the directed migration of microglia toward the injury site, spatial concentration gradients of chemoattractant molecules are generated (Fan et al., 2017; Lively and Schlichter, 2018; Omar Zaki et al., 2019). In our study, migration was stimulated with 10% serum. The results showed increased migratory capacity of microglia in the presence of serum. However, no significant differences in microglia migration were observed between isolation protocols. Nevertheless, cells isolated with PROTOCOL 1 showed a tendency for better migration.

Reactive oxygen species are a hallmark of neurodegeneration and contribute to disease progression (Simpson and Oliver, 2020). Microglial dysfunction is primarily associated with increased production of ROS, which causes DNA damage (von Bernhardi et al., 2015). Activated microglia can also overproduce prostaglandins, chemokines, cytokines, and reactive oxygen and nitrogen species, which can impair neuron survival by increasing oxidative stress and activating cell death pathways (Redza-Dutordoir and Averill-Bates, 2016). It has been shown that treatment with LPS, the major constituents of Gram-negative bacteria, increases intracellular ROS production in microglia *in vitro* in a dose-dependent manner (Wang et al., 2004). In this study, a significant increase in ROS production was observed in all microglia cultures tested compared to the control cells when microglia were exposed to an LPS stimulus. Nevertheless, no difference was observed between isolation protocols in both treated and untreated cells. Therefore, we demonstrated that the isolation procedure did not affect the formation of ROS in primary microglia.

Studying the aging of microglia is key to understanding the cause of neurodegenerative diseases (Caldeira et al., 2014). Accumulation of senescent microglia with age has been demonstrated both in *in vivo* and *in vitro* models. Microglia are known to age faster and die rapidly in a single-cell microenvironment (Angelova and Brown, 2019). In culture, microglia that age exhibit a senescence phenotype, including increased SA-β-gal activity (Trias et al., 2019). To determine whether the isolation procedure affects microglia aging, cells were initially maintained in culture for 7 days, and no significant signs of senescence were observed. However, 1-month-old microglia showed a steep aging progression and subsequently higher activity of SA-β-gal. Isolation protocols showed no significant difference at either time point. However, we recommend performing experiments related to functionality within 2 weeks of early senescence.

## Conclusion

We found no significant difference between the isolation protocols used in this study in terms of cell purity, functionality, and aging. These data suggest that microglia do not change their dynamic behavior to a more irresponsive phenotype depending on the cell isolation method. Therefore, all three protocols can be used in the study of neuroinflammatory responses under different pathological conditions to distinguish microglia-driven responses. However, the yield was significantly higher with our modified PROTOCOL 1. In research where they require close to 100% purity, we recommend perfusion of the brain tissue to eliminate any other cell type which may contaminate the purity of culture. In addition, there was a difference in the procedure length of each protocol, an important factor to consider.

We have provided three total-length protocols with all precautions and steps so that readers can choose the protocol that best suits their research needs and available laboratory equipment (as described in Supplementary material, with all precautions to address issues that may arise when isolating microglia).

However, *in vitro* microglia cannot be maintained in culture for extended periods of time. Therefore, although the proposed protocol extends the life of primary microglia in culture, it should be noted that microglia isolated from adult mouse brain behave differently outside the organism and that in *in vitro* studies should be carefully considered.

## Data availability statement

The raw data supporting the conclusions of this article will be made available by the authors, without undue reservation.

## Ethics statement

The animal study was reviewed and approved by the Lithuanian State Food and Veterinary Service (No. G2–104).

## Author contributions

DB, VM, and AB designed the experiments and revised the manuscript. AV and MI performed the experiments and data analysis and wrote the manuscript. AB conceived the project. All authors contributed to the article and approved the submitted version.

## References

[B1] Agalave N. M. Lane B. T. Mody P. H. Szabo-Pardi T. A. Burton M. D. (2020). Isolation, culture, and downstream characterization of primary microglia and astrocytes from adult rodent brain and spinal cord. *J. Neurosci. Methods* 340:108742. 10.1016/J.JNEUMETH.2020.108742 32315669 PMC7293863

[B2] Aktories P. Petry P. Kierdorf K. (2022). Microglia in a Dish—Which techniques are on the menu for functional studies? *Front. Cell. Neurosci.* 16:908315. 10.3389/FNCEL.2022.908315/FULL PMC9204042 35722614

[B3] Angelova D. M. Brown D. R. (2019). Microglia and the aging brain: Are senescent microglia the key to neurodegeneration? *J. Neurochem.* 151 676–688. 10.1111/JNC.14860 31478208

[B4] Avazzadeh S. Dehkordi M. H. Owens P. Jalali A. Coffey K. Fernhead H. O. (2022). Establishing electroporation thresholds for targeted cell specific cardiac ablation in a 2D culture model. *J. Cardiovasc. Electrophysiol.* 33 2050–2061. 10.1111/jce.15641 35924470 PMC9543844

[B5] Bennett M. L. Bennett F. C. (2020). The influence of environment and origin on brain resident macrophages and implications for therapy. *Nat. Neurosci.* 23 157–166. 10.1038/S41593-019-0545-6 31792468

[B6] Bohlen C. J. Bennett F. C. Tucker A. F. Collins H. Y. Mulinyawe S. B. Barres B. A. (2017). Diverse requirements for microglial survival, specification, and function revealed by defined-medium cultures. *Neuron* 94 759–773.e8. 10.1016/J.NEURON.2017.04.043 28521131 PMC5523817

[B7] Bokarewa M. Rodas L. Martínez S. Riera-Sampol A. Moir H. J. Tauler P. (2021). Blood cell *in vitro* cytokine production in response to lipopolysaccharide stimulation in a healthy population: Effects of age, sex, and smoking. *Cells* 11:103. 10.3390/cells11010103 35011664 PMC8750398

[B8] Bordt E. A. Block C. L. Petrozziello T. Sadri-Vakili G. Smith C. J. Edlow A. G. (2020). Isolation of Microglia from mouse or human tissue. *STAR Protoc.* 1:100035. 10.1016/J.XPRO.2020.100035 32783030 PMC7416840

[B9] Brás J. P. Bravo J. Freitas J. Barbosa M. A. Santos S. G. Summavielle T. (2020). TNF-alpha-induced microglia activation requires miR-342: Impact on NF-kB signaling and neurotoxicity. *Cell Death Dis.* 11:415. 10.1038/s41419-020-2626-6 32488063 PMC7265562

[B10] Buenaventura R. G. Harvey A. C. Burns M. P. Main B. S. (2022). Sequential isolation of microglia and astrocytes from young and aged adult mouse brains for downstream Transcriptomic analysis. *Methods Protoc.* 5:77. 10.3390/mps5050077 36287049 PMC9610580

[B11] Butovsky O. Jedrychowski M. P. Moore C. S. Cialic R. Lanser A. J. Gabriely G. (2014). Identification of a Unique TGF-β Dependent Molecular and Functional Signature in Microglia. *Nat. Neurosci.* 17 131–143. 10.1038/NN.3599 24316888 PMC4066672

[B12] Caldeira C. Oliveira A. F. Cunha C. Vaz A. R. Falcão A. S. Fernandes A. (2014). Microglia change from a reactive to an age-like phenotype with the time in culture. *Front. Cell. Neurosci.* 8:152. 10.3389/FNCEL.2014.00152/ABSTRACT 24917789 PMC4040822

[B13] Candlish M. Hefendehl J. K. (2021). Microglia phenotypes converge in aging and neurodegenerative disease. *Front. Neurol.* 12:660720. 10.3389/FNEUR.2021.660720 34025562 PMC8133315

[B14] Cardona A. E. Huang D. R. Sasse M. E. Ransohoff R. M. (2006). Isolation of murine microglial cells for RNA analysis or flow cytometry. *Nat. Protoc.* 1 1947–1951. 10.1038/nprot.2006.327 17487181

[B15] Ceyzériat K. ben Haim L. Denizot A. Pommier D. Matos M. Guillemaud O. (2018). Modulation of astrocyte reactivity improves functional deficits in mouse models of Alzheimer’s disease. *Acta Neuropathol. Commun.* 6:104. 10.1186/S40478-018-0606-1 30322407 PMC6190663

[B16] Chen A. Leith M. Tu R. Tahim G. Sudra A. Bhargava S. (2017). Effects of diluents on cell culture viability measured by automated cell counter. *PLoS One* 12:e0173375. 10.1371/journal.pone.0173375 28264018 PMC5338812

[B17] Crain J. M. Nikodemova M. Watters J. J. (2013). Microglia express distinct M1 and M2 phenotypic markers in the postnatal and adult central nervous system in male and female mice. *J. Neurosci. Res.* 91 1143–1151. 10.1002/JNR.23242 23686747 PMC3715560

[B18] Fan Y. Xie L. Chung C. Y. (2017). Signaling pathways controlling microglia chemotaxis. *Mol. Cells* 40 163–168. 10.14348/molcells.2017.0011 28301917 PMC5386953

[B19] Fernández-Arjona M. D. M. Grondona J. M. Granados-Durán P. Fernández-Llebrez P. López-Ávalos M. D. (2017). Microglia morphological categorization in a rat model of neuroinflammation by Hierarchical cluster and principal components analysis. *Front. Cell. Neurosci.* 11:235. 10.3389/FNCEL.2017.00235 28848398 PMC5550745

[B20] Floden A. M. Combs C. K. (2007). Microglia repetitively isolated from in vitro mixed glial cultures retain their initial phenotype. *J. Neurosci. Methods* 164 218–224. 10.1016/J.JNEUMETH.2007.04.018 17553568 PMC2041803

[B21] Frank M. G. Wieseler-Frank J. L. Watkins L. R. Maier S. F. (2006). Rapid isolation of highly enriched and quiescent microglia from adult rat hippocampus: Immunophenotypic and functional characteristics. *J. Neurosci. Methods* 151 121–130. 10.1016/J.JNEUMETH.2005.06.026 16125247

[B22] Garcia J. A. Cardona S. M. Cardona A. E. (2014). Isolation and analysis of mouse Microglial cells. *Curr. Protoc. Immunol.* 104 Unit–14.35. 10.1002/0471142735.IM1435S104 24510618 PMC3980480

[B23] Gerrits E. Heng Y. Boddeke E. W. G. M. Eggen B. J. L. (2020). Transcriptional profiling of microglia; Current state of the art and future perspectives. *Glia* 68 740–755. 10.1002/GLIA.23767 31846124 PMC7064956

[B24] Gingras M. Gagnon V. Minotti S. Durham H. D. Berthod F. (2007). Optimized protocols for isolation of primary motor neurons, astrocytes and microglia from embryonic mouse spinal cord. *J. Neurosci. Methods* 163 111–118. 10.1016/J.JNEUMETH.2007.02.024 17445905

[B25] Ginhoux F. Greter M. Leboeuf M. Nandi S. See P. Gokhan S. (2010). Fate mapping analysis reveals that adult microglia derive from primitive macrophages. *Science* 330 841–845. 10.1126/SCIENCE.1194637 20966214 PMC3719181

[B26] Giulian D. Baker T. J. (1986). Characterization of ameboid microglia isolated from developing mammalian brain. *J. Neurosci.* 6 2163–2178. 3018187 10.1523/JNEUROSCI.06-08-02163.1986 PMC6568755

[B27] Glenn J. A. Ward S. A. Stone C. R. Booth P. L. Thomas W. E. (1992). Characterisation of ramified microglial cells: Detailed morphology, morphological plasticity and proliferative capability. *J. Anat.* 180 109–118. 1452465 PMC1259614

[B28] Gordon R. Hogan C. E. Neal M. L. Anantharam V. Kanthasamy A. G. Kanthasamy A. (2011). A simple magnetic separation method for high-yield isolation of pure primary microglia. *J. Neurosci. Methods* 194 287–296. 10.1016/J.JNEUMETH.2010.11.001 21074565 PMC3205446

[B29] Grabert K. McColl B. W. (2018). Isolation and phenotyping of adult mouse microglial cells. *Methods Mol. Biol.* 1784 77–86. 10.1007/978-1-4939-7837-3_7 29761389

[B30] Gyoneva S. Orr A. G. Traynelis S. F. (2009). Differential regulation of microglial motility by ATP/ADP and adenosine. *Parkinsonism Relat. Disord.* 15(Suppl. 3), S195–S199. 10.1016/S1353-8020(09)70813-2 20082989 PMC8867539

[B31] Hanamsagar R. Bilbo S. D. (2017). Environment matters: Microglia function and dysfunction in a changing world. *Curr. Opin. Neurobiol.* 47 146–155. 10.1016/J.CONB.2017.10.007 29096243 PMC5732848

[B32] Harry G. J. Kraft A. D. (2012). Microglia in the developing brain: A potential target with lifetime effects. *Neurotoxicology* 33 191–206. 10.1016/J.NEURO.2012.01.012 22322212 PMC3299893

[B33] Helmut K. Hanisch U. K. Noda M. Verkhratsky A. (2011). Physiology of microglia. *Physiol. Rev.* 91 461–553. 10.1152/PHYSREV.00011.2010/SUPPL_FILE/VIDEO2.AVI 21527731

[B34] Hickman S. Izzy S. Sen P. Morsett L. el Khoury J. (2018). Microglia in neurodegeneration. *Nat. Neurosci.* 21 1359–1369. 10.1038/s41593-018-0242-x 30258234 PMC6817969

[B35] Holland R. McIntosh A. L. Finucane O. M. Mela V. Rubio-Araiz A. Timmons G. (2018). Inflammatory microglia are glycolytic and iron retentive and typify the microglia in APP/PS1 mice. *Brain Behav. Immun.* 68 183–196. 10.1016/J.BBI.2017.10.017 29061364

[B36] Hovens I. Nyakas C. Schoemaker R. (2014). A novel method for evaluating microglial activation using ionized calcium-binding adaptor protein-1 staining: Cell body to cell size ratio. *Neuroimmunol. Neuroinflamm.* 1:82. 10.4103/2347-8659.139719

[B37] Jolivel V. Brun S. Binamé F. Benyounes J. Taleb O. Bagnard D. (2021). Microglial cell morphology and phagocytic activity are critically regulated by the neurosteroid allopregnanolone: A possible role in neuroprotection. *Cells* 10:698. 10.3390/cells10030698 33801063 PMC8004004

[B38] Law A. M. K. Rodriguez de la Fuente L. Grundy T. J. Fang G. Valdes-Mora F. Gallego-Ortega D. (2021). Advancements in 3D cell culture systems for personalizing anti-cancer therapies. *Front. Oncol.* 11:782766. 10.3389/FONC.2021.782766/FULL PMC8669727 34917509

[B39] Lee J. K. Tansey M. G. (2013). Microglia isolation from adult mouse brain. *Methods Mol. Biol.* 1041 17–23. 10.1007/978-1-62703-520-0_3 23813365 PMC4145600

[B40] Leyh J. Paeschke S. Mages B. Michalski D. Nowicki M. Bechmann I. (2021). Classification of microglial morphological phenotypes using machine learning. *Front. Cell. Neurosci.* 15:241. 10.3389/FNCEL.2021.701673/BIBTEX PMC8276040 34267628

[B41] Ling E. -A. Wong W. -C. (1993). The origin and nature of ramified and amoeboid microglia: A historical review and current concepts. *Glia* 7 9–18. 10.1002/GLIA.440070105 8423067

[B42] Lively S. Schlichter L. C. (2018). Microglia responses to pro-inflammatory stimuli (LPS, IFNγ+TNFα) and reprogramming by resolving cytokines (IL-4, IL-10). *Front. Cell. Neurosci.* 12:215. 10.3389/FNCEL.2018.00215/BIBTEX PMC6066613 30087595

[B43] Lizana-Vasquez G. D. Arrieta-Viana L. F. Mendez-Vega J. Acevedo A. Torres-Lugo M. (2022). Synthetic thermo-responsive terpolymers as tunable scaffolds for cell culture applications. *Polymers* 14:4379. 10.3390/polym14204379 36297960 PMC9611013

[B44] Luan W. Li M. Wu C. Shen X. Sun Z. (2022). Proteomic dissimilarities of primary microglia and BV2 cells under stimuli. *Eur. J. Neurosci.* 55 1709–1723. 10.1111/EJN.15637 35239205

[B45] Mcintosh A. Mela V. Harty Conor Minogue A. M. Costello D. A. (2019). Iron accumulation in microglia triggers a cascade of events that leads to altered metabolism and compromised function in APP/PS1 mice. *Brain Pathol.* 29 606–621. 10.1111/bpa.12704 30661261 PMC8028264

[B46] Megur A. Baltriukien D. Bukelskien V. Burokas A. (2020). The Microbiota-Gut-Brain Axis and Alzheimer’s Disease: Neuroinflammation Is to Blame? *Nutrients* 13:37. 10.3390/nu13010037 33374235 PMC7824474

[B47] Miyoshi H. Abo K. Hosoya D. Matsuo K. Utsumi Y. (2022). Effects of mouse fetal liver cell culture density on hematopoietic cell expansion in three-dimensional cocultures with stromal cells. *Int. J. Artif. Organs* 45 103–112. 10.1177/0391398821996377 33611956

[B48] Montilla A. Zabala A. Matute C. Domercq M. (2020). Functional and metabolic characterization of microglia culture in a defined medium. *Front. Cell Neurosci.* 14:22. 10.3389/fncel.2020.00022 32116565 PMC7025516

[B49] Muhammad M. (2019). “Tumor necrosis factor alpha: A major cytokine of brain neuroinflammation,” in *Cytokines*, ed. Behzadi P. (London: IntechOpen). 10.5772/INTECHOPEN.85476

[B50] Ni M. Aschner M. (2010). Neonatal rat primary microglia: Isolation, culturing and Selected applications. *Curr. Protoc. Toxicol.* CHAPTER, Unit–12.17. 10.1002/0471140856.TX1217S43 20960423 PMC2959194

[B51] Nikodemova M. Watters J. J. (2012). Efficient isolation of live microglia with preserved phenotypes from adult mouse brain. *J. Neuroinflammation* 9:147. 10.1186/1742-2094-9-147 22742584 PMC3418565

[B52] Omar Zaki S. S. Kanesan L. Leong M. Y. D. Vidyadaran S. (2019). The influence of serum-supplemented culture media in a transwell migration assay. *Cell Biol. Int.* 43 1201–1204. 10.1002/CBIN.11122 30811086

[B53] Paolicelli R. C. Sierra A. Stevens B. Tremblay M. E. Aguzzi A. Ajami B. (2022). Microglia states and nomenclature: A field at its crossroads. *Neuron* 110 3458–3483. 10.1016/J.NEURON.2022.10.020 36327895 PMC9999291

[B54] Parkhurst C. N. Gan W. B. (2010). Microglia dynamics and function in the CNS. *Curr. Opin. Neurobiol.* 20 595–600. 10.1016/J.CONB.2010.07.002 20705452 PMC3708473

[B55] Perry S. W. Dewhurst S. Bellizzi M. J. Gelbard H. A. (2002). Tumor necrosis factor-alpha in normal and diseased brain: Conflicting effects via intraneuronal receptor crosstalk? *J. Neurovirol.* 8 611–624. 10.1080/13550280290101021 12476354 PMC7094979

[B56] Podolnikova N. P. Kushchayeva Y. S. Wu Y. F. Faust J. Ugarova T. P. (2016). The role of integrins αMβ2 (Mac-1, CD11b/CD18) and αDβ2 (CD11d/CD18) in macrophage fusion. *Am. J. Pathol.* 186 2105–2116. 10.1016/J.AJPATH.2016.04.001 27315778 PMC4973655

[B57] Redza-Dutordoir M. Averill-Bates D. A. (2016). Activation of apoptosis signalling pathways by reactive oxygen species. *Biochim. Biophys. Acta Mol. Cell Res.* 1863 2977–2992. 10.1016/J.BBAMCR.2016.09.012 27646922

[B58] Rubio-Araiz A. Finucane O. M. Keogh S. Lynch M. A. (2018). Anti-TLR2 antibody triggers oxidative phosphorylation in microglia and increases phagocytosis of β-amyloid. *J. Neuroinflammation* 15:247. 10.1186/s12974-018-1281-7 30170611 PMC6119264

[B59] Saijo K. Glass C. K. (2011). Microglial cell origin and phenotypes in health and disease. *Nat. Rev. Immunol.* 11 775–787. 10.1038/nri3086 22025055

[B60] Simpson D. S. A. Oliver P. L. (2020). Ros generation in microglia: Understanding oxidative stress and inflammation in neurodegenerative disease. *Antioxidants* 9:743. 10.3390/ANTIOX9080743 32823544 PMC7463655

[B61] Stansley B. Post J. Hensley K. (2012). A comparative review of cell culture systems for the study of microglial biology in Alzheimer’s disease. *J. Neuroinflammation* 9 1–8. 10.1186/1742-2094-9-115/TABLES/2 22651808 PMC3407712

[B62] Tang Y. Le W. (2016). Differential roles of M1 and M2 microglia in neurodegenerative diseases. *Mol. Neurobiol.* 53 1181–1194. 10.1007/S12035-014-9070-5 25598354

[B63] Telpoukhovskaia M. A. Liu K. Sayed F. A. Etchegaray J. I. Xie M. Zhan L. (2020). Discovery of small molecules that normalize the transcriptome and enhance cysteine cathepsin activity in progranulin-deficient microglia. *Sci. Rep.* 10:13688. 10.1038/s41598-020-70534-9 32792571 PMC7426857

[B64] Trias E. Beilby P. R. Kovacs M. Ibarburu S. Varela V. Barreto-Núñez R. (2019). Emergence of microglia bearing senescence markers during paralysis progression in a rat model of inherited ALS. *Front. Aging Neurosci.* 10:42. 10.3389/FNAGI.2019.00042/FULL PMC6403180 30873018

[B65] von Bernhardi R. Eugenín-von Bernhardi L. Eugenín J. (2015). Microglial cell dysregulation in brain aging and neurodegeneration. *Front. Aging Neurosci.* 7:124. 10.3389/FNAGI.2015.00124/ABSTRACT 26257642 PMC4507468

[B66] Wang T. Qin L. Liu B. Liu Y. Wilson B. Eling T. E. (2004). Role of reactive oxygen species in LPS-induced production of prostaglandin E2 in microglia. *J. Neurochem.* 88 939–947. 10.1046/J.1471-4159.2003.02242.X 14756815

[B67] Wang Y. Szretter K. J. Vermi W. Gilfillan S. Rossini C. Cella M. (2012). IL-34 is a tissue-restricted ligand of CSF1R required for the development of Langerhans cells and microglia. *Nat. Immunol.* 13 753–760. 10.1038/NI.2360 22729249 PMC3941469

[B68] Watson P. M. D. Kavanagh E. Allenby G. Vassey M. (2017). Bioengineered 3D glial cell culture systems and applications for neurodegeneration and neuroinflammation. *SLAS Discov.* 22 583–601. 10.1177/2472555217691450 28346104

[B69] Wei Z. Sun T. Shimoda S. Chen Z. Chen X. Wang H. (2022). Bio-inspired engineering of a perfusion culture platform for guided three-dimensional nerve cell growth and differentiation †. *Lab Chip* 22:1006. 10.1039/d1lc01149a 35147637

[B70] Woolf Z. Stevenson T. J. Lee K. Jung Y. Park T. I. H. Curtis M. A. (2021). Isolation of adult mouse microglia using their *in vitro* adherent properties. *STAR Protoc.* 2:100518. 10.1016/J.XPRO.2021.100518 34027479 PMC8121984

