# Supplementary material for: Microglia isolation from aging mice for cell culture: A beginner’s guide
Source: Front Cell Neurosci. 2023 Jan 19;17:1082180. doi: 10.3389/fncel.2023.1082180 (PMC9893793; doi:10.3389/fncel.2023.1082180)
Supplement: Supplementary file 1 [file Data_Sheet_1.pdf]

Microglia isolation from aging mice for cell culture: a beginner's guide

Akshay Kumar Vijaya [1,†], Monika Iešmantaitė [1,†], Virginia Mela [2], Daiva Baltriukienė [1], Aurelijus Burokas [1]

Supplementary material

Supplementary Materials and Methods

Transfer buffer

Add 5 mL DMEM/F-12 media in a 15 mL tube + 400 µL of 10,000 U/mL of penicillin/streptomycin. Prepare fresh before use and keep on ice.

**Volumes adjusted for 1 brain tissue*

Enzyme Digestion Mix

PROTOCOL 1

In a 15 mL tube, add 10 mg Collagenase A (0.197 U/mg; Roche, Switzerland) + 70 µL DNase I (1000 U/mL; Ref:10104159001; Roche, Switzerland) + 50 µL HEPES (1M; Thermo Fisher Scientific, Lithuania) + 250 µL FBS (Ref: A3840401; Thermo Fisher Scientific, Lithuania) + HBSS ((without calcium and magnesium) to a final volume of 5 mL (Ref:14175-095; Thermo Fisher Scientific, Lithuania). Initially, incubate at room temperature for 30 minutes, followed by +4°C for another 30 minutes.

**Volumes adjusted for 1 brain tissue*

PROTOCOL 2

Add protease to 5 mL HBSS (20U/ml) and gently mix to dissolve. Add 8.65 mL of DMEM/F-12 media + 1.25 mL dissolved protease (Ref: P0652; Sigma, USA) and 100 µL of DNase (1000 U/mL). Warm to 37°C before use.

**Volumes adjusted for 1 brain tissue*

PROTOCOL 3

Dilute the HEPES-buffered saline containing the lyophilized dispase II (Ref: D4693; Sigma, United States) with a cell culture medium to obtain a final concentration of 2.4 U/mL. In a 15 mL tube. Prepare Dispase DNase Papain (DDP) solution. 0.028 g papain (Ref: 10108014001; Sigma, USA) to a final concentration of 1 mg/mL + 14 mL of DMEM/F12 + 14 mL premade

dispase II (final concentration 1.2 U/mL). Store at -20°C. Add DNAse I (final concentration 20 U/mL) right before use.

Isolations

PROTOCOL 1

Although numerous protocols have been developed to isolate cells from young mice, the isolation of adult mouse microglia has proven difficult till the present time. The cellular heterogeneity of the brain makes it technically very challenging to obtain sufficient and pure microglia for *in vitro* culture. Studying and understanding microglia for age-related diseases requires the protocol to be simple and consistent to generate healthy cells from adult or aged mice.

We present a modified protocol (redesigned (Bordt et al., 2020)) to isolate pure primary microglia for various downstream applications. We furnished a simple, widely accessible protocol. This technique utilizes dissociated brain tissue from adult mice to produce mixed glial cell cultures. After 3 hours, primary microglia are obtained by replacing the media with floating cells, leaving behind pure microglia. The microglia are then plated for experimental study relating to functional assays.

1. Dice the brain (not perfused) with scissors/scalpel into small pieces.
2. Transfer to enzyme digestion mix (pipette and mix well).
3. Incubate at 37°C for 15 minutes. Shake gently every 5 minutes. Agitate the tubes but do not invert to mix, to prevent the tissue from being trapped on the lid of the 15 mL tube.
4. Over the course of three sequential flame-polished Pasteur pipets, dissociate tissue to a single-cell solution (firstly use 7 mm, then 5 mm, and lastly 3mm Pasteur pipets). With each pipet, pipet up and down 20 times. Between each numbered pipet stage, incubate for 15 minutes at 37°C.
5. Filter through sterile 70 µm nylon filter.
6. Add HBSS (with calcium and magnesium) (Ref:14025-092; Thermo Fisher Scientific, Lithuania) up to 14 mL.
7. Centrifuge for 10 minutes at 300 × g, 4°C, max acceleration, half brake.
8. Discard the supernatant and slowly resuspend the pellet with 3100 µL of 1x PBS.

9. Add 900 µL of Debris Removal Solution (Ref:5201108766; Miltenyi Biotec, Germany), pipette gently up and down ten times.
10. Slowly layer 4 mL of 1x PBS on top. Centrifuge for 10 minutes, max speed (at least 3000 × g), 4°C, max acceleration, max break.
11. Remove the top two layers.
12. Add up to 14 mL of 1x PBS, centrifuge for 10 minutes, at 1000 × g, 4°C, max acceleration, max break.
13. Remove the supernatant.
14. Resuspend cells in 300 µL of Red Blood Cells Lysis Buffer (Ref: R7757; Sigma, USA), and incubate for 5 minutes at room temperature.
15. Add 5 mL of cell media or PBS, centrifuge for 5 minutes, 20°C, 200 × g.
16. Discard supernatant.
17. Add 5mL complete media (DMEM/F-12 (1:1) (1X) + GlutaMAX (Ref:31331-028; Thermo Fisher Scientific, Lithuania) with 10% Fetal bovine serum (FBS) and 1% penicillin/streptomycin (10,000 units) and seed in T25 flask.
18. After 3 hours, discard media (containing unattached cells) and add new media. If needed, add 50% conditioned media supplement.
19. The next day add GM-CSF and M-CSF to a final concentration of 100 ng/mL. These growth factors will support the cells to proliferate and obtain their specific morphology.
20. Maintain cells for 7 days and monitor cell growth.

PROTOCOL 2

Isolation was performed as described in the paper (Woolf et al., 2021). The adherent property of microglia was exploited and paved the way for this new technique, which allows users to isolate cells without much difficulty. This technique utilizes dissociated brain tissue from adult mice to produce mixed glial cell cultures. After the mixed glial cultures are seeded for 6 hours, primary microglia are mechanically isolated from the culture by a brief duration of tapping. Microglia can be cultured and used as a model to study age-related diseases.

1. Dice the unperfused tissue into small cubes < 1 mm^3 using a sterile scalpel.
2. Add 10 mL of enzyme digestion mixture to the diced tissue in the Petri dish. Collect the tissue fragments media and transfer it to the sterile Falcon tube.
3. Incubate for 15 minutes at 37°C. Agitate every 5 minutes.

4. Triturate the mixture ∼20 times till large clumps of tissue are removed and re-incubate for a further 15 minutes at 37°C. Agitate every 5 minutes.
5. Triturate the mixture a further ∼10 times.
6. Pass the digested tissue through a sterile 70 µm Nylon mesh filter into a sterile 50 mL Falcon tube. Pass a further 10 mL of complete warm media through the filter to rinse, making a final volume of 20 mL.
7. Centrifuge the cell suspension at 170 × *g* for 5 minutes. Remove the supernatant.
8. Add complete media, and transfer into a non-coated T25 culture flask.
9. Wait for 6 hours for microglia to adhere to the flask.
10. Tap the flask gently 40 times to dissociate non-microglial cells.
11. Remove media containing floating non-adherent cells, discard, and replace with fresh, warmed complete media.
12. Check for any adherent cells which are not microglia (non-phase bright cells) under the microscope. Remove all media containing floating non-adherent cells, discard, and replace with complete fresh media.
13. The next day, add GM-CSF and M-CSF to a 100 ng/mL final concentration. Maintain cells for 7 days and monitor cell growth.

PROTOCOL 3

Isolation was performed as described in the paper (Lee and Tansey, 2013). Isolation of microglia from adult mouse brains is quite complex, and percoll provides us with an alternative method to isolate microglia and remains a very reliable way to do the same. Homogenized brain tissue may be separated by density gradient centrifugation to yield primary microglia. Here we describe a refined protocol including enzymatic and mechanical dissociation of adult mouse brain tissue and myelin removal by percoll density gradient.

The cells are isolated in a very healthy state and allow the user to conduct experiments related to functional assays, immunocytochemistry, and flow cytometry analysis. In addition, Percoll is an efficient tool with a low-viscosity, non-toxic medium suitable for density gradient centrifugation of cells.

1. Cut the unperfused brain with scissors/scalpel into small cubes 1 mm^3 using a sterile scalpel in 2 mL of transfer media.
2. Transfer the minced brain to a 15 mL tube containing 3 mL dissociation medium.

3. Gently rock (or invert the tube every 5 minutes) cell suspension in a tissue culture
1. incubator for 20 minutes.
4. Neutralize the enzymes in dissociation media by adding 5 mL of neutralization media.
5. Centrifuge 5 minutes at 250×g at room temperature.
6. Aspirate the media slowly (be extremely cautious not to disturb the pellet as it can
2. easily be re-aspirated by the aspirating pipet).
7. Resuspend the pellet in 5 mL of serum-free media. Repeat steps 5 and 6.
8. Add 3 mL DMEM/F12 and pipette up and down with polished large 7 mm size Pasteur pipette against the bottom of the tube until large clumps of tissue are broken up.
9. Add 3 mL of DMEM/F12 and pipette up and down with polished medium 5 mm size Pasteur pipette against the bottom of the tube until large clumps of tissue are broken up.
10. Add 2 mL DMEM/F12 and pipette up and down with polished small 3 mm size Pasteur pipette to break clumps.
11. Wet 40 μm cell strainer with 2 mL of DMEM/F12 and filter cell suspension through a cell strainer.
14. Resuspend cells in 5 mL DMEM/F12, spin 250 × g for 4 minutes, and remove supernatant.
15. Resuspend cell pellet in 4 mL per brain of 37% stock isotonic Percoll (SIP) (Ref: P4937; Sigma, USA).
16. Transfer 4 mL of the 37% SIP (from Step 15) to 15 mL conical tubes and slowly underlay 4 mL of 70% SIP. Then on top of the 37% layer, slowly pipette 4 mL of 30% SIP, followed by 2 mL of HBSS.
17. Centrifuge gradient 40 minutes at 300 × g (18°C) with no brake.
    *Important!! Make sure to use no brake so that the interphase is not disturbed.
18. Using a transfer pipette, gently remove a layer of debris and collect 2.0–2.5 mL of the 70 -37% interphase into a clean 15 mL conical tube.
19. Add 6 mL of HBSS for each 2 mL of the interphase volume collected to ensure the percoll containing the interphase is diluted about three times.
20. Centrifuge 7 minutes at 500 × g at 4°C.
21. Resuspend pellet in 500 μL of HBSS and transfer to small 0.6 mL or 1.5 mL tubes and wash 3 times in a volume of 500 μL, using a microcentrifuge at 800 × g at 4°C.
22. Count cells using a hemocytometer.
23. The next day, add GM-CSF and M-CSF to a final concentration of 100 ng/mL.

24. Maintain cells for seven days and monitor cell growth.

Preparation of conditioned media

All floating cells isolated at the end of PROTOCOL 1 were seeded in a 75 cm^2 flask in complete media (DMEM/F-12 (1:1) (1X) + GlutaMAX). The cells were allowed to proliferate till confluency, followed by media collection, and new media was added. The collected media was passed through a 0.2 μm filter to remove any cells and debris. The filtered media was later mixed in equal parts with complete media (DMEM/F-12 (1:1) (1X) + GlutaMAX). The conditioned media is ready to use. * Possible not to use and substituted by regular (DMEM/F-12 (1:1) (1X) + GlutaMAX.

CAUTIONS

- Counting cells after isolation for PROTOCOL 1 and PROTOCOL 2 will be difficult because the cell density also consists of other cell types, debris, and dead cells, as shown in (**Figure S1**). Therefore, the data in this article only includes the total number of cells after 7 days.

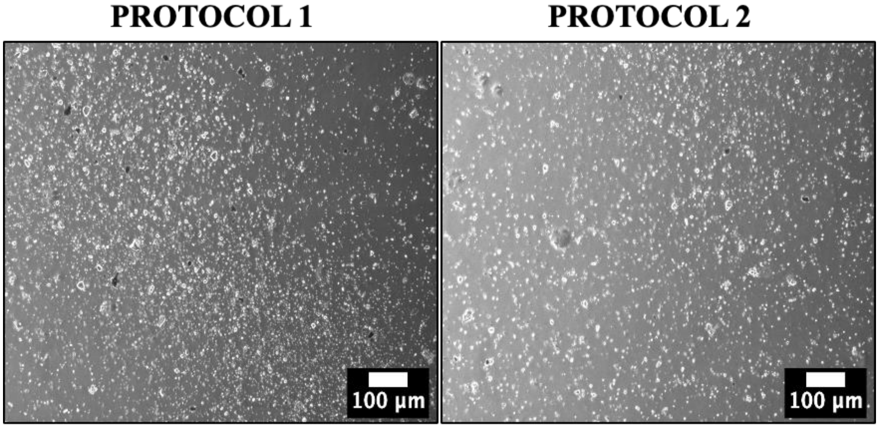

FIGURE S1. Visible debris after isolation from PROTOCOL1 and PROTOCOL 2 makes it difficult to count cell post isolation.

- The most crucial part of microglia isolation using percoll is forming the density gradient. 70% SIP solution should be underlaid very slowly, avoiding mixing the phases. If phases are disturbed, it will result in a lower yield and purity of microglial cells. The improper layering of Percoll with different concentrations will lead to poor density gradient and eventual improper separation of cells **(Figure S2)**. A good density gradient of Percoll concentration should have myelin floating at the top, microglia at

the interphase, and red blood cells at the bottom. Also, it is preferable to use high-quality, pure Percoll to get the best results.

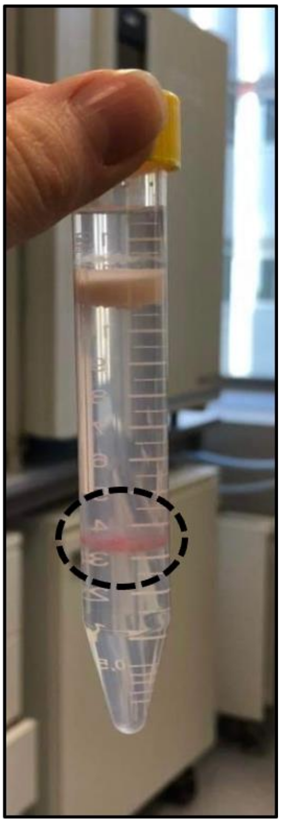

FIGURE S2. Improper percoll layering at step 16 in PROTOCOL 3 (Figure 1) due to poor handling or improper preparation of SIP. Presence of RBCs at microglia gradient (highlighted by black ring).

- Microglia isolation using their *in vitro* adherent properties isolation protocol suggests tapping the flask vigorously after 6-8 hours after seeding. However, while adapting this protocol in our laboratory, we noticed that gentle tapping 30-40 times is sufficient to detach unwanted cells. Also, vigorous tapping will detach microglia cells and reduce cell yield **(Figure S3)**. Moreover, it seems that vigorous tapping exposes microglia to stress and can result in cell activation.

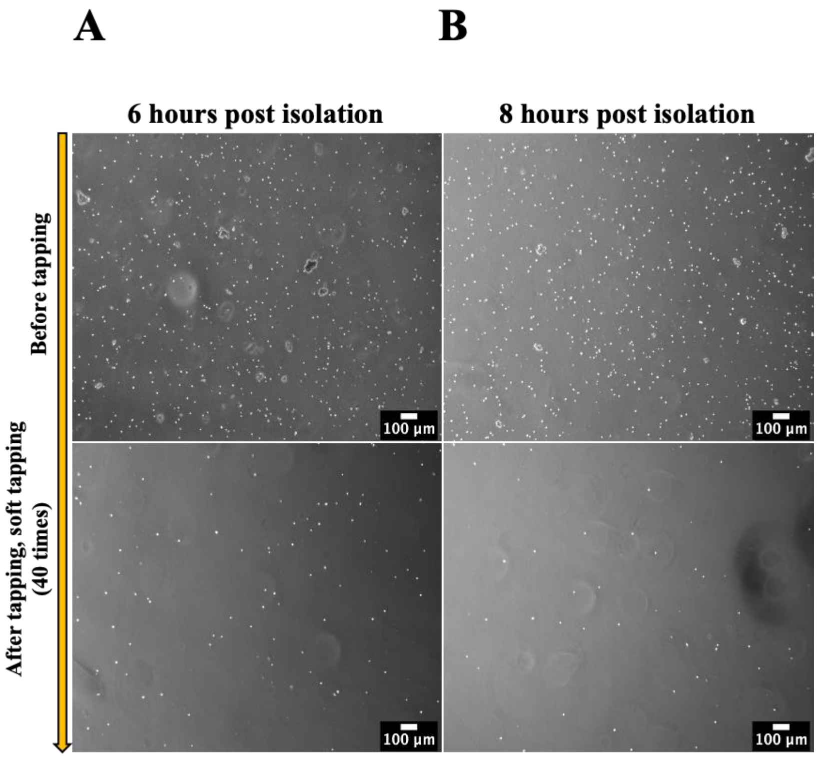

FIGURE S3. Post isolation step for PROTOCOL 2. **A.** shows cells that remain after tapping to detach unwanted cells after 6 hrs. **B.** shows cells that remain after tapping to detach unwanted cells after 8 hrs.

- While using PROTOCOL 1 for microglia isolation, it is essential to layer the PBS on top of the cells suspended in PBS and debris removal solution carefully and very slowly. A poorly made gradient will dissociate the myelin throughout the PBS and leave behind cell debris, which will affect the future steps as shown in **(Figure S4)**. It is also advised to use a swinging bucket for this centrifugation steps.

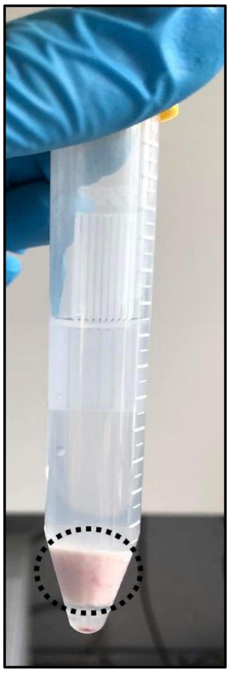

FIGURE S4. Improper PBS layering at step 12 in PROTOCOL 1 (Figure 1) (highlighted by black ring).

- Experiments should be conducted on ice, and buffers should be chilled when it is advised according to protocols. A low temperature will slow metabolic activities and prevent biochemical or molecular changes that may affect microglia activation. It helps to minimize *ex vivo* changes in microglial gene expression.

- Magnetic bead separation: With our protocol and reagents, combining them with magnetic beads is unsuitable for isolation. These methods obtain cells but fail to attach and form morphology even after ten days. The flasks were also coated with poly-d-lysine for better adhesion. Nevertheless, similar results were observed, as shown in **(Figure 5)**. Some papers use magnetic beads to their advantage (Sarkar et al., 2019) and the instruments used for their protocol were obtained from the company "MILTENYL BIOTEC".

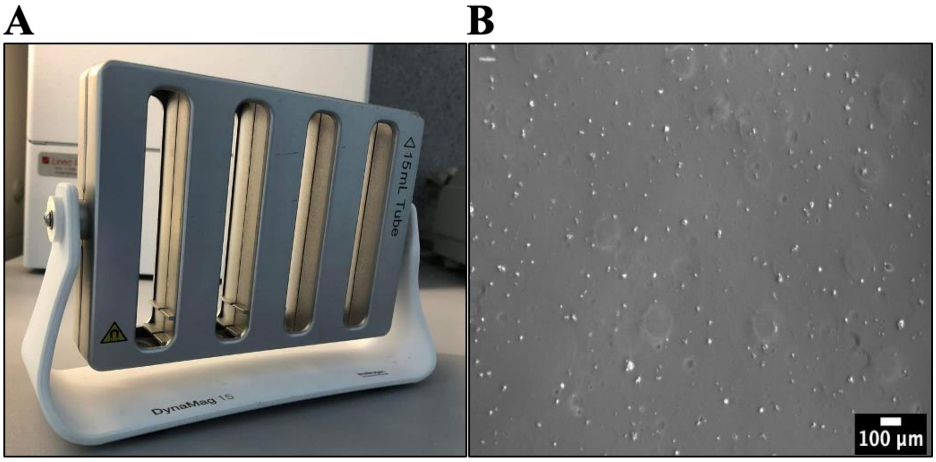

FIGURE S5. Magnetic bead isolation. **A.** Magnetic bead separator used by our team (Ref no: 10266783; ThermoFisher Scientific, Lithuania). **B.** Potential microglia cells separated using magnetic bead separator as mentioned above. Unfortunately, these cells failed to attach even after 10 days due to the blockage of the CD11b receptor as CD11b microbeads were used to separate the cells. Evidence shows that this receptor is essential for cell adhesion (Podolnikova et al., 2016).

Supplementary References

Bordt, E. A., Block, C. L., Petrozziello, T., Sadri-Vakili, G., Smith, C. J., Edlow, A. G., et al. (2020). Isolation of Microglia from Mouse or Human Tissue. *STAR Protoc* 1. doi: 10.1016/J.XPRO.2020.100035.

Lee, J. K., and Tansey, M. G. (2013). Microglia isolation from adult mouse brain. *Methods Mol. Biol.* 1041, 17–23. doi: 10.1007/978-1-62703-520-0_3.

Podolnikova, N. P., Kushchayeva, Y. S., Wu, Y. F., Faust, J., and Ugarova, T. P. (2016). The Role of Integrins αMβ2 (Mac-1, CD11b/CD18) and αDβ2 (CD11d/CD18) in Macrophage Fusion. *Am. J. Pathol.* 186, 2105–2116. doi: 10.1016/J.AJPATH.2016.04.001.

Sarkar, S., Harishchandra, D. S., Ghaisas, S., Panicker, N., Charli, A., Palanisamy, B. N., et al. ARTICLE Mitochondrial impairment in microglia amplifies NLRP3 inflammasome proinflammatory signaling in cell culture and animal models of Parkinson’s disease. *NPJ Parkinsons Dis.* 3:30. doi: 10.1038/s41531-017-0032-2.

Woolf, Z., Stevenson, T. J., Lee, K., Jung, Y., Park, T. I. H., Curtis, M. A., et al. (2021). Isolation of adult mouse microglia using their in vitro adherent properties. *STAR Protoc* 2. doi: 10.1016/J.XPRO.2021.100518.
